# Community Assembly Processes of Deadwood Mycobiome in a Tropical Forest Revealed by Long-Read Third-Generation Sequencing

**DOI:** 10.1007/s00248-024-02372-5

**Published:** 2024-05-03

**Authors:** Witoon Purahong, Li Ji, Yu-Ting Wu

**Affiliations:** 1 Department of Soil Ecology, UFZ-Helmholtz Centre for Environmental Research, Theodor-Lieser-Str. 4, 06120 Halle (Saale), Germany; 2 School of Forestry, Central South University of Forestry and Technology, Changsha, 410004 China; 3 Department of Forestry, National Pingtung University of Science and Technology, Pingtung, 91201 Taiwan; 4 Department of Biomedical Science and Environmental Biology, Kaohsiung Medical University, Kaohsiung, 80708 Taiwan

**Keywords:** Deadwood mycobiome, Long-read third-generation sequencing, Tropical forests

## Abstract

Despite the importance of wood-inhabiting fungi on nutrient cycling and ecosystem functions, their ecology, especially related to their community assembly, is still highly unexplored. In this study, we analyzed the wood-inhabiting fungal richness, community composition, and phylogenetics using PacBio sequencing. Opposite to what has been expected that deterministic processes especially environmental filtering through wood-physicochemical properties controls the community assembly of wood-inhabiting fungal communities, here we showed that both deterministic and stochastic processes can highly contribute to the community assembly processes of wood-inhabiting fungi in this tropical forest. We demonstrated that the dynamics of stochastic and deterministic processes varied with wood decomposition stages. The initial stage was mainly governed by a deterministic process (homogenous selection), whereas the early and later decomposition stages were governed by the stochastic processes (ecological drift). Deterministic processes were highly contributed by wood physicochemical properties (especially macronutrients and hemicellulose) rather than soil physicochemical factors. We elucidated that fine-scale fungal-fungal interactions, especially the network topology, modularity, and keystone taxa of wood-inhabiting fungal communities, strongly differed in an initial and decomposing deadwood. This current study contributes to a better understanding of the ecological processes of wood-inhabiting fungi in tropical regions where the knowledge of wood-inhabiting fungi is highly limited.

**Supplementary Information:**

The online version contains supplementary material available at 10.1007/s00248-024-02372-5.

## Introduction

Deadwood serves as an important carbon and nutrient source that is difficult to assess by most organisms [1]. Wood-inhabiting fungi play important roles in the decay and mineralization processes of deadwood which alter the complex substrates into various forms of available nutrients to render them accessible to other organisms [2]. Main deadwood mycobiome can be categorized into white-rot, brown-rot, and soft-rot fungi which generally co-occur in nature, targeting at more or less different niches and components of deadwood (e.g., cellulose, hemicellulose, pectin, and lignin) [3]. Bacteria also co-occur with wood-inhabiting fungi and facilitate the deadwood decay processes by providing additional N [4]. Some bacteria are found to directly decompose complex polymers in wood, such as cellulose, hemicellulose, and even lignin [5]. Nevertheless, a recent study found that most (~ 90% or more) of identified peptides of deadwood have a fungal origin which infers that fungi are the main producers of extracellular enzymes in deadwood [6]. Despite the importance of wood-inhabiting fungi on nutrient cycling and many ecosystem functions, their ecological processes are still not fully understood [7, 8]. While the majority of studies on wood-inhabiting fungal communities focus on the deterministic process to disentangle the relative effects of different abiotic factors shaping their community [2, 9, 10], we do not know how much deterministic processes contribute to the community assembly processes of wood-inhabiting fungi [7]. Furthermore, it is urgently needed to consider other ecological processes, including biotic filtering, dispersal, and stochastic processes which may play equal or even more important roles in the community assembly processes of wood-inhabiting fungi [7]. Deterministic and stochastic processes can occur simultaneously for shaping a given wood-inhabiting fungal community and the relative roles of each process may depend on the considered spatial scale [7]. Although forest management strategies, especially thinning activities, have been reported to influence the richness and/or community composition of wood-inhabiting fungal communities [11], the mechanisms on which processes drive such responses are still unclear. Applications of microbial ecological theories can enable us to better understand relevant factors related to organization, structure, and mechanistic insights of the assembly processes governing the wood-inhabiting fungal communities [12].

Deterministic processes are comprised of both homogeneous and variable selections in which abiotic and biotic factors play roles in filtering wood-inhabiting fungi suitable for specific environments. Abiotic environmental filtering is a part of the deterministic processes which has been well studied for wood-inhabiting fungi [2, 10, 13–16]. Different abiotic factors from small (e.g., deadwood level) to large scales can prevent or facilitate the establishment or persistence of different wood-inhabiting fungal species [7, 10]. At the deadwood level, wood pH, moisture content, wood macronutrients (i.e., C, N, P), micronutrients, and lignin content are important determinants [9, 10, 16]. High or low pH and moisture content facilitate different taxa and functional groups of wood-inhabiting fungi, specifically soft-rot fungi show tolerance to wide ranges of pH conditions and moisture contents exceed those of brown-rot and white-rot fungi [3, 17]. In general, wood-inhabiting fungi have optimal growth rates in the pH range of 3 to 6 (brown-rot fungi have the lowest optimal pH of  3 to 3.5 while white-rot fungi have been found to prefer a pH range of 4 to 4.5) [17]. For moisture content, wood-inhabiting fungi need moisture content above the fiber-saturation point of the wood (mostly ~ 25%); thus, free water can become available on the wood cell walls’ surface [17, 18]. However, wood decay can also occur at lower moisture content than the fiber-saturation point for wood of some tree species for specific fungal taxa [19]. In general, the optimum moisture content level for wood-inhabiting fungi ranges from 40 to 80% but white-rot fungi seem to prefer higher moisture content than brown-rot fungi [3]. High lignin content facilitates the white-rot fungi with ability for oxidative enzyme production [14]. Part of wood-inhabiting fungal communities arises from soil fungal communities [20]. Soil physicochemical parameters such as pH and macronutrients co-shape soil- and wood-inhabiting fungal communities [20]. Biotic filtering both intraspecific and interspecific interactions is not well understood for wood-inhabiting fungi. Intraspecific interactions are defined as density dependence whereas interspecific interactions are characterized as predation, mutualism, facilitation, and competition [7]. Competition is the most common and relatively well studied as compared with facilitation, mutualism, and predation [21]. Assembly history or priority effect may also be considered part of biotic filtering in which the initial community determines the latter communities [22]. This process is driven by interaction among wood-inhabiting fungi, especially competition [22, 23]. Deadwood harbors diverse organisms, thus apart from wood-inhabiting fungi bacteria and insects may play roles in biotic filtering [24, 25]. Facilitation and mutualism may be illustrated and analyzed by co-occurrence network patterns [25].

Stochastic processes refer to processes that generate divergence among communities sharing identical environments [7]. These processes include homogenizing dispersal, dispersal limitation, and undominated processes. Dispersal refers to both contemporary small-scale and large-scale migrations [7]. Undominated processes refer to demographic stochasticity, colonization-extinction stochasticity, environmental stochasticity, and ecological drift [7].

So far, wood-inhabiting fungal communities have been efficiently characterized using short-read next-generation sequencing technologies such as 454 pyrosequencing, ion torrent, and Illumina sequencing [9, 25]. These approaches are indeed useful to investigate the changes in wood-inhabiting fungal communities; however, the short-sequence reads generated by these sequencing technologies (cover either only fugal ITS1 or ITS2 regions) have been criticized for the accuracy of taxonomic assignments [26]. The choice of ITS region for metabarcoding has been also debated for decades [27]. Recently, long-read third-generation sequencing technologies have been developed and established [28]. Due to the longer sequence reads generated, many recent studies demonstrate that long-read third-generation sequencing technologies are more accurate for taxonomic assignment and phylogenetic analysis as compared with short-read next-generation sequencing technologies [29]. The sequences of complete ITS regions can be obtained from amplicon sequencing of the long-read third-generation technologies.

This current study aimed to use PacBio long-read third-generation sequencing to investigate the relative importance of community assembly processes of two deadwood mycobiomes in a tropical forest using the baiting technique. Specifically, we (i) tested the priority effects on wood-inhabiting fungal communities, (ii) quantified the relative importance of deterministic and stochastic process in shaping wood-inhabiting fungal communities along different sampling times over 2 years using beta nearest taxon index (BetaNTI), (iii) studied the interactions between wood-inhabiting fungi using co-occurrence network approach, (iv) studied the impacts of forest thinning activity levels on richness and community composition of wood-inhabiting fungal communities, and (v) determined the significant factors shaping wood-inhabiting fungal communities. We hypothesized that priority effects play an important role in structuring wood-inhabiting fungal communities by significantly determining the later community composition. We also hypothesized that deterministic processes are increasingly important during the decomposition process as soil- and wood-physicochemical properties change and interact with wood-inhabiting fungal communities over time [2, 30]. We expected that the fungal richness reduced with high thinning intensity as well as the changes in fungal community composition. Long-read third-generation sequencing approaches have been demonstrated to give more accurate taxonomic and functional information than short-read next-generation sequencing. Combining PacBio as the long-read third-generation sequencing and microbial assembly theory, our study represents the first study that quantifies the relative importance of deterministic and stochastic processes in shaping wood-inhabiting fungal communities in highly unexplored tropical forest ecosystems.

## Materials and Methods

### Study Site, Experimental Design and Sampling

The study was conducted on a 1-ha permanent site at Tajen Forest Station (22°27′N 120°82′E) in southeastern Taiwan. The forest at this site has been classified as a tropical forest. The site’s mean annual temperature and humidity are 25 °C and 75%, respectively, with a mean annual precipitation of 2400 mm (based on data from 1998 to 2006), falling mostly between April and October. A long-term experiment has been established at the 1-ha site, which has been divided into 20 plots subjected to five different thinning regimes (0, 20, 40, 60, and 80% cutting with four replicates for each) since February 2015; 1 plot equals to 20 × 25 m^2^ in area. The 1-ha study site harbors 83 vascular plant species, representing 31 families and 61 genera belonging to *Pasanio shinsuiensis*-*Castanopsietum carlesii* ass. Nov. hoc loco [31]. *Quercus pachyloma* and *Machilus thunbergii* as the two dominant tree species of plant community in the study site were chosen as the target species. The standardized fresh logs (ca. 50 cm long and 10–15-cm diameter) of *Q. pachyloma* and *M. thunbergii* were collected and randomly laid on the forest floor in each plot with three replicates, which in a total of 120 logs.

Sampling was conducted three times, at the initial stage of decomposition, and followed by 1-year and 2-year decay, respectively. Decay class was determined according to a four-decomposition-stage classification system [32]. Wood samples were collected using a cordless drill (Makita DDF 453) equipped with a wood auger (diameter 20 mm, length 135 mm), which was flamed and wiped with ethanol between drillings to avoid cross-contamination between samples. More than three wood holes were randomly drilled on the up-side of each log. Wood samples from each log were pooled, resulting in three composite samples of each wood species from each plot. The samples were frozen in liquid nitrogen immediately after collection, transported to the laboratory on dry ice (ca. − 70 °C) as soon as possible, and stored at − 20 °C until analysis. Soil samples (0–5-cm depth) were also collected from each plot where the logs were placed for soil physicochemical property analysis.

### Wood and Soil Physicochemical Properties

In this study, the equilibrium wood moisture content (EMC), mass losses (MLs), wood density, wood pH, cellulose content, hemicellulose content, Klason lignin, % ash of wood, total C, and total N were measured. Briefly, the equilibrium moisture content (EMC) of both the heat-treated samples (105 °C) and the untreated control samples were determined according to the Chinese National standard for the determination of the moisture content for physical and mechanical tests (CNS452). The wood mass losses (MLs) caused by the heat treatment were determined based on the oven-dried mass of the sample before treatment. The national standard of the determination of density for physical and mechanical tests (CNS451) was applied to determine the densities of the heat-treated and control samples. Wood pH values were determined by shaking 1 g of dried wood in 10 mL of distilled water for 120 min and measuring the pH of the resulting aqueous extract. Celluloses (holocellulose and α-cellulose contents) were measured acceding to CNS 4713, CNS 3085, and CNS 10865 methods. Hemi-cellulose contents were measured according to Zhao et al. (2010) [33]. Acid-insoluble lignin (Klason lignin) content was measured according to CNS 2721. Total carbon (C) and nitrogen (N) was measured by dry combustion at 1000 ℃ with a CHNS-Elemental Analyzer (Elementar Analysensysteme GmbH, Hanau, Germany). The ash content was determined using CNS 3084 method. Soil pH was determined using a WTW Multi 3510 IDS portable meter (Weilheim, Germany). Soil total nitrogen was analyzed using the Semimacro Kjeldahl method [34] while soil organic matter using the Walkley–Black procedure [35]. Soil available phosphorus was extracted and measured according to Bray 1 method [36], and exchangeable cations (K^+^, Mg^2+^, Ca^2+^, and Na^+^) [37] were by atomic absorption spectrophotometry using a Z 5300 instrument following recommendations of the manufacturer (Hitachi—Science & Technology, Tokyo, Japan). Soil water content was also measured. Detailed protocols of all wood and soil physicochemical analyses are provided in Supplementary Information.

### DNA Extraction, ITS Amplicon Library Generation, and Long-Read Third-Generation Sequencing

Each composite wood sample was separately cut into small pieces and homogenized into a fine powder. DNAs were extracted from 100 mg of each homogenized composite wood sample using ZR Soil Microbe DNA MiniPrep kit (Zymo Research, Irvine, CA, USA), according to the manufacturer’s instructions. The presence and quantity of genomic DNA were checked using a NanoDrop ND-1000 spectrophotometer (Thermo Fisher Scientific, Dreieich, Germany), and the extracts were then stored at − 20 °C. Fungal communities were characterized based on full-length ITS sequences. Amplicon libraries were generated using the fungus-specific universal primer pair ITS1F/ITS4. Triplicate PCR amplifications of each sample were performed in a total volume of 50 ml containing 1 × Taq Master Mix (Genechain Industrial Co., Ltd., Kaohsiung City, Taiwan), 0.2 µM of each primer, and 2–5 µg of diluted template DNA (final concentration, 100 ng). A touchdown PCR program was used involving an initial denaturation period (95 ℃ for 5 min) followed by 10 cycles of denaturation at 94 ℃ for 30 s, annealing at 60–50 ℃ for 45 s (1 ℃ per cycle), and extension at 72 ℃ for 2 min; and then 30 cycles of 94 ℃ for 30 s, 50 ℃ for 45 s, and 72 ℃ for 2 min, with a final 10 min extension step at 72 ℃. The PCR products were separated by 1% agarose gel electrophoresis using 1 × TAE buffer and SYBRR Green I. Bands of the expected size (between 500 and 1000 bp) were cut out and purified using a QIAEX II Gel Extraction Kit (QIAGEN Inc., Valencia, USA). Sixteen nucleotide barcoded SMRTbell adapters were added to the 50 ends of amplicons and reverse complement barcoded adapters were added to the 30 ends to enable multiplexing of samples. The barcoded samples were pooled at equal molar concentrations and multiplexed on single SMRT chips. Sequences were generated using the PacBio Sequel system, which can achieve 99.5–99.9% sequence accuracy for DNA fragments using circular consensus technology [38], at Genomics BioSci & Tech Co., Ltd. in Taiwan.

### Bioinformatics

Amplicon read processing was performed in R [39]. Fungal ITS region amplicons were sequenced by PacBio circular consensus sequencing (CCS) using Sequel systems, and subjected to DADA2 analysis [40]. Firstly, fungal primers for ITS region (ITS1F, 5′-CTTGGTCATTTAGAGGAAGTAA-3′; ITS4, 5′-TCCTCCGCTTATTGATATGC-3′) were truncated from CCS reads using the function remove primers. Following that, reads were quality filtered to remove those < 450 or > 1000 bp in length, containing any ambiguous base or harboring an expected error over 5. The sample inference was as default performed without “pseudo-pooling.” Amplicon sequence variants (ASVs) were taxonomically annotated as default with 50% probability cut-off by using UNITE v8.2 general release database (10.15156/BIO/786368). Such relatively lax confidence threshold of 50% was selected in order to increase the amount of input for consensus algorithm as explained in previous study [41]. The fungal ecological function of each ASV was determined using FungalTraits [42, 43]. The sequence reads of all samples were normalized to 1019 reads and used for further analyses. The fungal ITS raw read sequence datasets were deposited at the National Center for Biotechnology Information (NCBI) Sequence Read Archive (SRA) under bioproject number PRJNA887894.

### Assembly Processes and Co-occurrence Network Analysis

Based on random matrix theory, the Molecular Ecological Network Analysis Pipeline (http://ieg4.rccc.ou.edu/mena/) was used to construct the taxonomic networks of wood-inhabiting fungi across varied sampling times in the *Q. pachyloma* and *M. thunbergii* plantations. Then, the same network size and an average number of links were used to generate 100 corresponding random networks. The differences between the empirical network and the random networks were analyzed by *Z*-test. The detailed information refer to the description of previous publications [44, 45]. The intra-module connectivity value (*Zi*) and inter-module connectivity value (*Pi*) for each node were used to identify the keystone taxa in the network, following simplified criteria: (i) peripheral nodes (*Zi* < 2.5, *Pi* < 0.62), which were almost always associated to nodes within their modules; (ii) connectors (*Zi* < 2.5, *Pi* > 0.62), which were highly linked to several modules; (iii) module hubs (*Zi* > 2.5, *Pi* < 0.62), which were highly connected to numerous microbes in their modules; and (iv) network hubs (*Zi* > 2.5, *Pi* > 0.62), which acted as both module hubs and connectors. All network graphs were visualized by Gephi (v0.9.2).

Based on the null model theory described earlier [46, 47], the beta nearest taxon index (βNTI) was used to quantify the relative importance of deterministic and stochastic processes in the community assembly of wood-inhabiting fungi. Full-length fungal ITS sequences were used to generate the phylogenetic trees for the calculation of βNTI. Although the 18S nuclear ribosomal small subunit rRNA gene (SSU) is more appropriate and commonly used in phylogenetic analysis as compared with the ITS, it has a lower number of hypervariable domains in fungi. Nevertheless, the full-length ITS lengths of at least 400 bp were found to be sufficient to infer the fungal phylogeny and identify their corrected taxonomic information [29, 48]. This current study focuses on both taxonomic identification and phylogenetic placement; thus, the full-length ITS gene was chosen. In addition to βNTI, the Raup-Crick (RC_bray_) null model based on Bray–Curtis dissimilarity was further used to quantify dispersal-based stochastic ecological processes generating the turnover of community composition [49]. The homogeneous and variable selection are indicated by βNTI <  − 2 and βNTI >  + 2, respectively. The relative importance of dispersal limitation and homogenizing dispersal processes were assessed by |βNTI|< 2 but RCbray >  + 0.95 and RCbray <  − 0.95, respectively, and the undominated process was estimated by |βNTI|< 2 and |RCbray|< 0.95 [49]. The βNTI values based on Bray–Curtis dissimilarity were calculated by “iCAMP” package in R with the code provided by Ning et al. (2020) [46] (https://github.com/DaliangNing/iCAMP1).

### Statistical Analysis

Factors shaping wood-inhabiting fungal richness were analyzed using multiple regression analyses in SPSS (IBM SPSS Statistics 22, New York, NY, United States). Factors corresponding with wood-inhabiting fungal communities in deadwood of the two tree species across different stages of decomposition and thinning intensity levels were analyzed using Non-metric Multidimensional Scaling (NMDS) and a goodness-of-fit statistics based on the Bray–Curtis distance measure (*P*-values being based on 999 permutations) [50] implemented in the “vegan” package in *R* [51]. The difference in decay class (decomposition stage) between the two deadwood species (*Q. pachyloma* and *M. thunbergii*) was compared using a two-sample *t-*test implemented in PAST [52]. Normality and equality of group variance for both datasets were tested using the Shapiro–Wilk W test and the *F* test in PAST software. Impacts of the community assembly history on fungal community composition in each tree species were assessed using the Mantel test in PAST software.

## Results

### General Overview of Mycobiome and Deadwood Decomposition

**Fig. 1:**
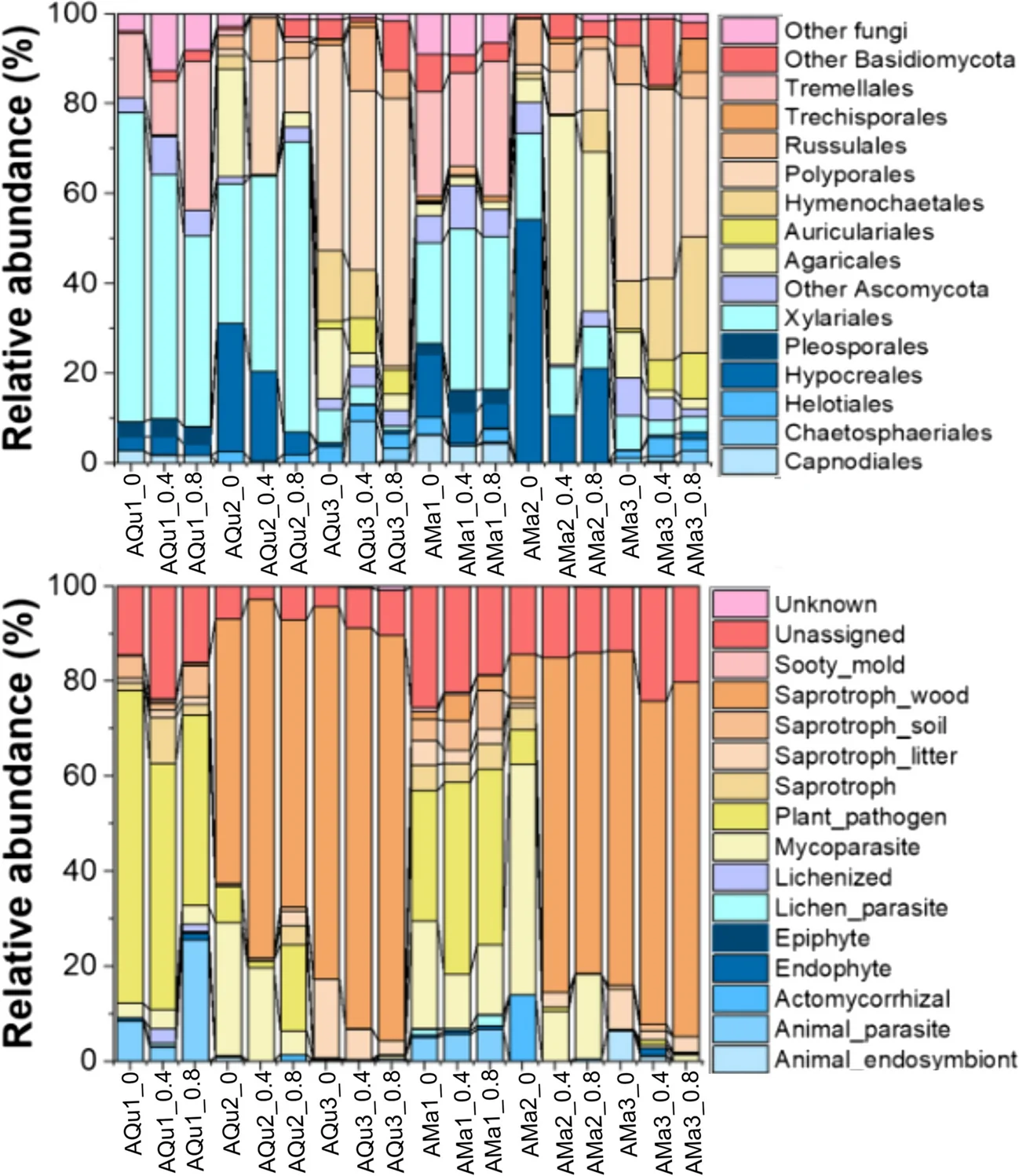
Average relative abundances of specific fungal taxonomic (orders) and functional groups of wood inhabiting fungi in deadwood of two tree species (*Quercus pachyloma* (Qu) and *Machilus thunbergii* (Ma)) at initial stage (AQu1 and AMa1), 1 year (AQu2 and AMa2), and 2 years (AQu3 and AMa3) with different level of thinning intensity (0 to 20% (0, non-thinning to low intensity treatment), 40 to 60% (0.4, intermediate intensity thinning treatment), and 80% (0.8, high intensity thinning treatment))

A total of 110,052 quality-filtered fungal ITS reads were obtained from 108 out of 120 wood samples after the removal of chimeric sequences and singletons and normalized to the smallest sequencing read number per sample (1019 sequences) (Table S1). The final analysis dataset contained 2207 fungal ASVs which were assigned to 71 orders and 272 genera (complete taxonomy provided in Tables S1 and S2, Supporting Information). There were 203 ASVs shared between the two tree species whereas 693 and 1311 ASVs specifically detected in *Q. pachyloma* and *M. thunbergii*, respectively. Xylariales (Ascomycota) and Tremellales (Basidiomycota) co-dominated the initial mycobiome of both tree species (Fig. 1). Xylariales were able to maintain their high relative abundances in *Q. pachyloma* after 1 year; however, Tremellales was replaced by Hypocreales and other members of Basidiomycota such as Agaricales, Polyporales, and Russulales. In 1-year *M. thunbergii* samples, Xylariales and Tremellales both strongly reduced in their relative abundances and were replaced by Hypocreales (non-thinning to low-intensity treatment) or Agaricales (thinning treatments) (Fig. 1). After 2 years, Polyporales and Hymenochaetales co-dominated the mycobiome of both tree species (except for the high thinning intensity of *Q. pachyloma* samples, only dominated by Polyporales).

The functional succession of wood-inhabiting fungi in both tree species was similar, changing from plant pathogens at the initial stages to wood saprotrophs and mycoparasite after 1 year and eventually highly dominated by wood saprotrophs after 2 years (Fig. 1). Thinning had minor impacts on functional composition of wood-inhabiting fungi in both tree species, specifically thinning intensity showed clear effect only in 1-year *M. thunbergii* samples which mycoparasite highly dominated non-thinning to low-intensity treatment, whereas wood saprotrophs highly dominated intermediate and high-intensity thinning treatments (Fig. 1). The successions of fungi with different decay types were also similar in both tree species, changing from soft-rot-dominated communities at the initial stages to soft- and white-rot communities after 1 year and finally to white-rot communities after 2 years (Tables S1 and S2). Brown-rot fungi mainly co-occurred with white-rot fungi in 2-year decomposing deadwood (Tables S1 and S2). After 2 years, the majority of deadwood from both tree species reached decay class 3 (intermediate decomposition stage, Fig S1). We detected no significant difference of decomposition stages between *Q. pachyloma* and *M. thunbergii* (*t* =  − 0.77, *P* = 0.448, Fig S1)*.*

### Assembly Processes: Stochastic vs. Deterministic Process

**Fig. 2:**
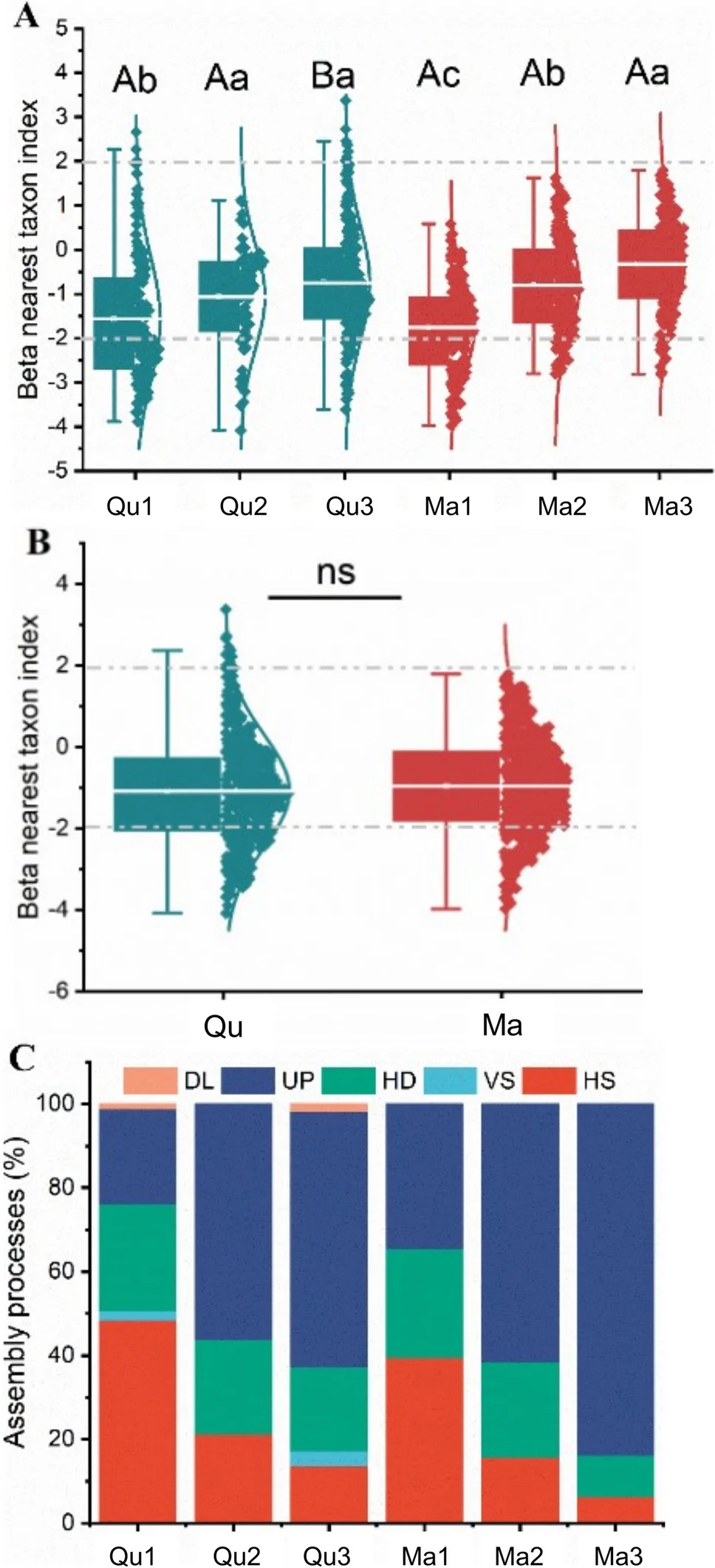
Contributions of stochastic vs. deterministic processes on wood-inhabiting fungal community assembly in deadwood of two tree species (*Quercus pachyloma* (Qu) and *Machilus thunbergii* (Ma)) at initial stage (Qu1 and Ma1), 1 year (Qu2 and Ma2), and 2 years (Qu3 and Ma3). **A** The betaNTI index between *Q. pachyloma* and *M. thunbergii* in three decomposition stages; **B** the betaNTI index between *Q. pachyloma* and *M. thunbergia*; **C** the ecological processes of wood-fungal community assembly. Upper letters indicate the significant difference between tree species; lower letters indicate the significant difference among time points byTurkey HSD test and *T* test, respectively. Processes: homogeneous selection (HS), variable selection (VS), homogenizing dispersal (HS), undominated processes (UP), and dispersal limitation (DL)

We detected dynamics of stochastic and deterministic processes which were changing with time (Fig. 2, Table S3). Homogenous selection (deterministic process) highly dominated the initial deadwood of both tree species and strongly reduced over time. In contrast, undominated processes (ecological drift) increase after deadwood incorporation into soils. In 2-year deadwood of *M. thunbergii*, undominated processes contributed 83.82% of wood-inhabiting fungal community assembly. Homogenizing dispersal was another stochastic process consistently shaping the wood-inhabiting fungal community assembly by contributing more than 20% (except for 2 years M*. thunbergii*, 9.86%). Variable selection (deterministic process, 2.21–3.68%) and dispersal limitation (stochastic process, 1.07–1.75%) contribute very little to the wood-inhabiting fungal community assembly in *Q. pachyloma* deadwood whereas they did not contribute at all in *M. thunbergii*.

### Fungal-Fungal Interactions in Deadwood Strongly Differ in an Initial and Decomposing Deadwood

**Fig. 3:**
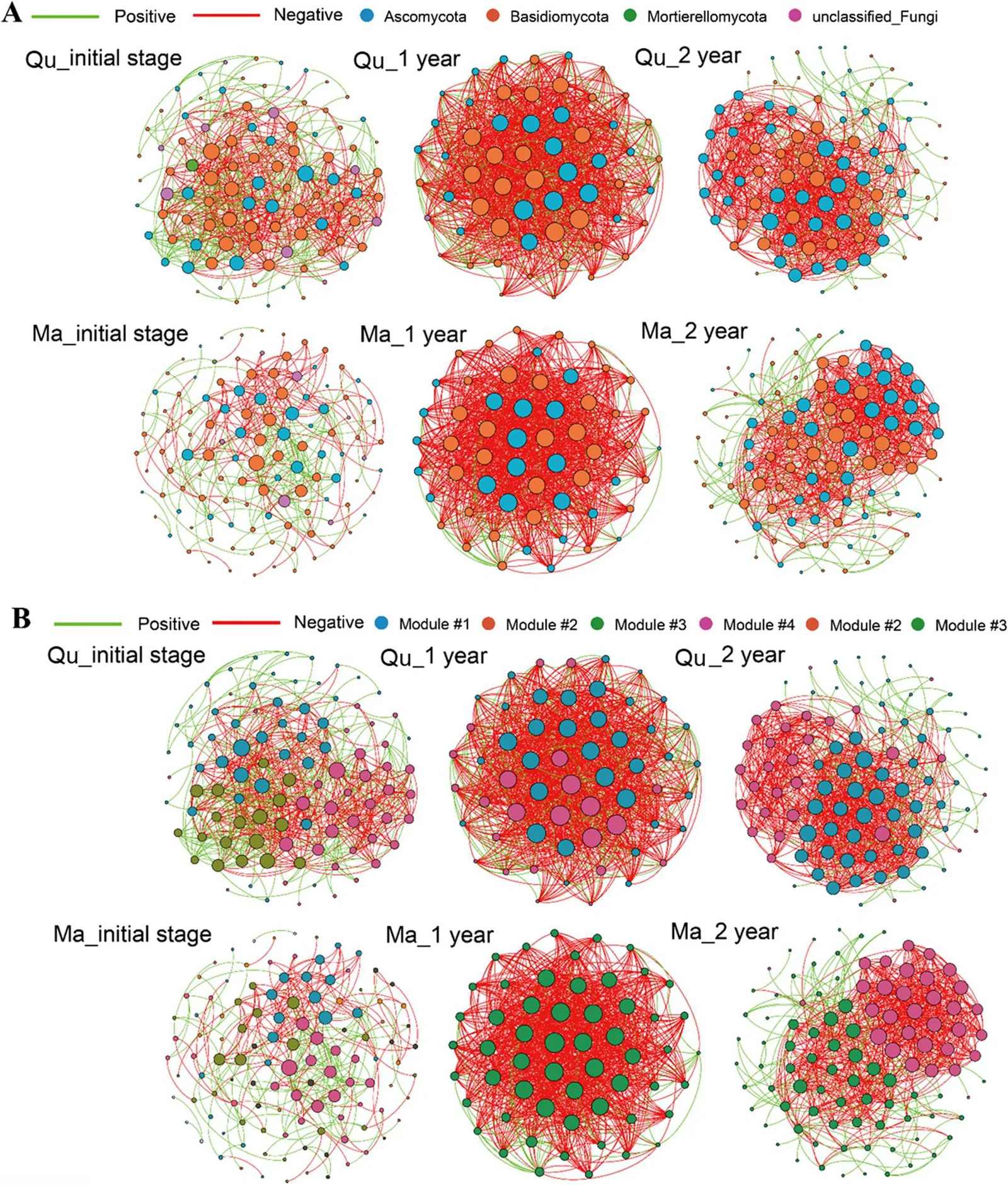
Taxonomic (**A**) and modular networks (**B**) of wood-inhabiting fungal communities in deadwood of two tree species (*Quercus pachyloma* (Qu) and *Machilus thunbergii* (Ma)) at different stages of decomposition

We observed the strong changes in network structure and topology between initial, 1-year, and 2-year decomposing deadwood and the results were consistent in both tree species tested in this experiment (Table 1 and Fig. 3). In both tree species, we observed the strong reduction of modularity and average path distance but an increase of clustering coefficient, average degree, modularity, and number of links (both positive and negative links) between co-occurrence networks of initial deadwood as compared with 1-year decomposing wood (Table 1). The co-occurrence network of 2-year decomposing deadwood exhibited higher modularity, number of positive correlations, and average path distance and lower number of negative links, average degree, and average clustering coefficient as compared with 1-year decomposing wood (Table 1).

**Table 1:** Topological properties of the networks of wood-inhabiting fungal communities in deadwood of two tree species (*Quercus pachyloma* (Qu) and *Machilus thunbergii* (Ma)) at different stages of decomposition

Network type	Network features	Qu_initial stage	Qu_1 year	Qu_2 year	Ma_initial stage	Ma_1 year	Ma_2 year
Empirical network	Number of nodes	96	58	95	114	56	99
	Number of links	618	999	784	307	916	711
	*R*^2^ of power-law	0.154	0.12	0.047	0.659	0.124	0.142
	Number of positive correlations	243 (60.1%)	498 (71.6%)	563 (77.7%)	243 (60.1%)	498 (71.6%)	563 (77.7%)
	Number of negative correlations	161 (39.9%)	198 (28.4%)	162 (22.3%)	161 (39.9%)	198 (28.4%)	162 (22.3%)
	Average degree (avgK)	12.875	34.448	17.231	5.386	32.714	14.364
	Average clustering coefficient (avgCC)	0.272	0.708	0.451	0.304	0.747	0.340
	Average path distance (GD)	2.382	1.396	2.281	4.105	1.405	2.406
	Modularity	0.339	0.055	0.209	0.573	0.005	0.333
Random network	avgCC ± SD	0.233 ± 0.015	0.729 ± 0.004	0.343 ± 0.018	0.087 ± 0.014	0.775 ± 0.005	0.291 ± 0.017
	GD ± SD	2.212 ± 0.019	1.396 ± 0.001	2.100 ± 0.016	2.990 ± 0.045	1.405 ± 0.001	2.206 ± 0.017
	Modularity ± SD	0.179 ± 0.008	0.041 ± 0.015	0.129 ± 0.008	0.359 ± 0.010	0.033 ± 0.014	0.156 ± 0.007

**Fig. 4:**
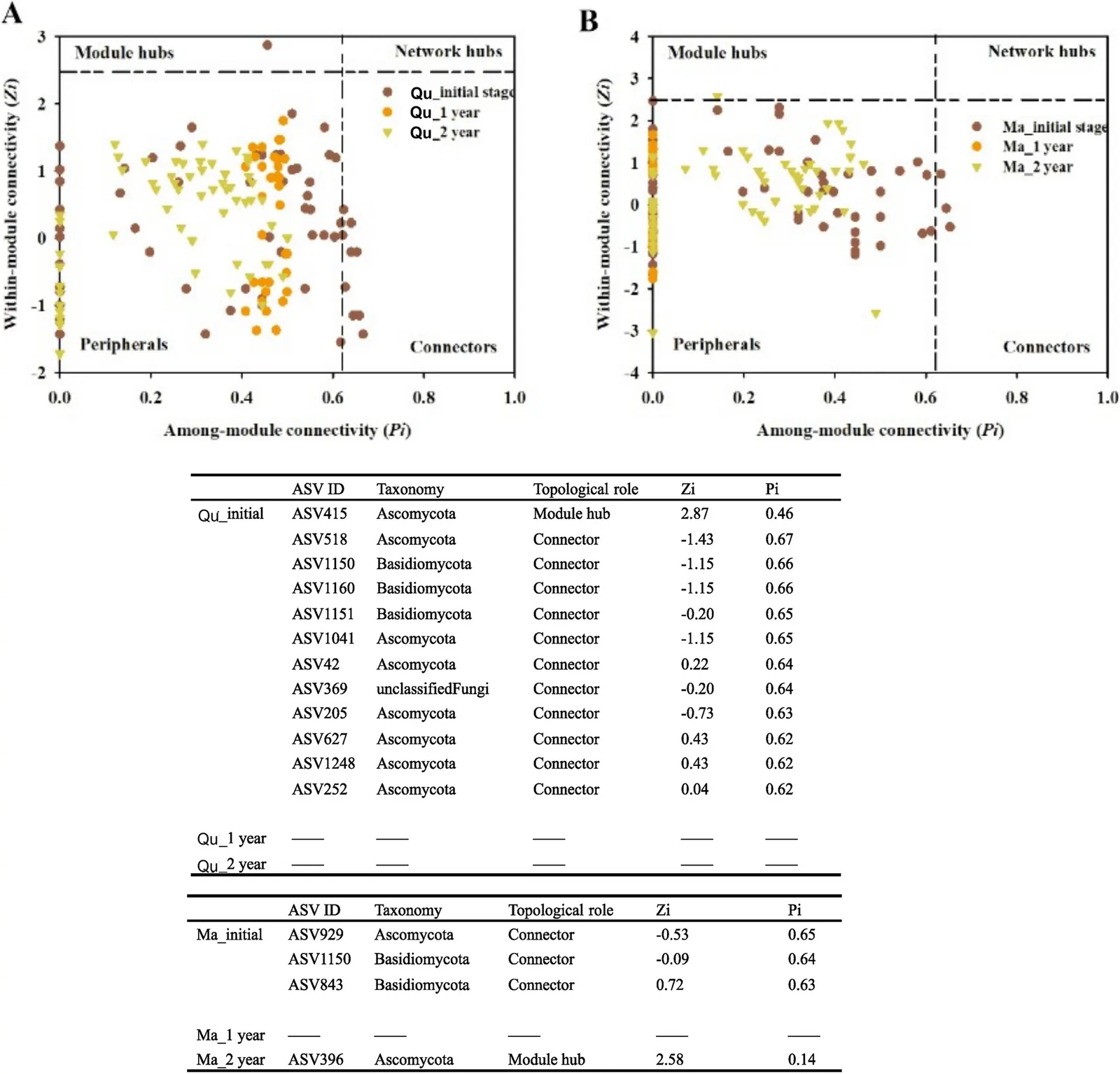
Keystone taxa of wood-inhabiting fungal communities in deadwood of two tree species at different stages of decomposition. **A** *Quercus pachyloma* (Qu); **B** *Machilus thunbergii* (Ma)

Based on the co-occurrence network, we identified the keystone taxa, including the module hub and connector. In total, we identified 12 taxa for *Q. pachyloma* (one module hub and 11 connectors) and four taxa for *M. thunbergii* (one module hub and three connectors). The module hub taxa were all Ascomycota whereas connectors include both Ascomycota and Basidiomycota as well as one taxon of unidentified fungi. The keystone taxa from the co-occurrence networks of the two tree species were mostly different. The only exception is for ASV1150 (belonging to Basidiomycota) which was identified as connectors in both tree species. Interestingly, the keystone taxa were mostly classified to have other functions (i.e., plant pathogen, mycoparasite, fungal decomposer) rather than wood decomposer (Fig. 4, Table S2).

### Factors Shaping Wood-Inhabiting Fungal Richness and Community Composition

 Tree species identity significantly affected the richness of wood-inhabiting fungi (*P* < 0.05); specifically, *M. thunbergii* had significantly higher wood-inhabiting fungal richness as compared with *Q. pachyloma*. Time series analysis showed that thinning intensity levels significantly impacted on the richness of wood-inhabiting fungi in *M. thunbergii* (*F* = 4.71, *P* = 0.025) but not in *Q. pachyloma* (*F* = 4.71, *P* = 0.025). Moderate thinning level significantly reduced the fungal richness as compared with control (no thinning activities) and high-intensity thinning activities. Tree species identity (*F* = 2.76, *P* = 0.001) and decomposition time (*F* = 7.53, *P* = 0.001) significantly determined wood-inhabiting fungal community composition. Thinning activity levels significantly shape the wood-inhabiting fungal community composition in *Q. pachyloma* (*R* = 0.18, *P* = 0.049). Furthermore, various wood-physicochemical properties, including extractives, holocellulose content, cellulose content, hemicellulose content, wood N content, wood C content, and wood pH significantly shaped wood-inhabiting fungal community composition in *Q. pachyloma*, whereas only extractives, hemicellulose content, and C content were significant factors in *M. thunbergii* (Table 2). It is noteworthy that all measured plot soil physicochemical properties (including macronutrient: P, K^+^, Ca^2+^, Mg^2+^, and N; micronutrient: Na^+^, soil organic matter, pH, and water content) were not significantly related to the wood-inhabiting fungal community composition in any of the tree species tested in this study (Table 2). We detected no significant impacts of the community assembly history in both tree species (*Q. pachyloma*: *R*_mantel_ = 0.10, *P* = 0.260; *M. thunbergii*:* R*_mantel_= 0.02, *P* = 0.581).

**Table 2:** Factors corresponding with wood-inhabiting fungal communities in deadwood of two tree species across different stages of decomposition and thinning intensity levels. Significant factors (*P* < 0.05) were indicated with bold *P* values.

Factors	*Quercus pachyloma*				*Machilus thunbergii*			
	**NMDS1**	**NMDS2**	***r***^**2**^	***P***	**NMDS1**	**NMDS2**	***r***^**2**^	***P***
Time	-0.56	0.83	0.2758	**0.009**	1.00	-0.10	0.669	**0.001**
Thinning	-0.79	0.61	0.1832	**0.049**	-0.92	-0.40	0.0327	0.545
Al_ben_extractives	-0.63	0.78	0.3149	**0.005**	0.95	-0.31	0.1777	**0.033**
Holocellulose	-0.93	0.38	0.3843	**0.001**	0.89	-0.45	0.1032	0.155
cellulose	-0.96	0.28	0.3755	**0.001**	-0.90	-0.44	0.0217	0.678
Hemicellulose	-0.82	0.57	0.1824	**0.046**	0.97	-0.24	0.411	**0.001**
Klason lignin	-0.70	0.71	0.0612	0.404	-0.52	-0.85	0.1372	*0.085*
Wood N content	-0.59	0.81	0.3413	**0.004**	0.92	-0.39	0.0537	0.362
Wood C content	-0.97	-0.24	0.3033	**0.005**	1.00	0.03	0.4544	**0.001**
Wood pH	0.64	-0.77	0.3164	**0.005**	-0.86	0.52	0.0254	0.645
Soil K	-0.83	0.56	0.0935	0.245	0.99	-0.16	0.0155	0.761
Soil Na	-0.71	0.71	0.0835	0.297	0.57	0.82	0.009	0.837
Soil Ca	-0.97	0.25	0.1497	0.101	-0.04	-1.00	0.0033	0.932
Soil Mg	-0.97	0.26	0.0788	0.306	-0.03	-1.00	0.0126	0.793
Soil organic matter	-0.53	0.85	0.0462	0.518	-0.48	-0.88	0.051	0.385
Soil P	0.67	0.74	0.1099	0.194	-0.34	-0.94	0.0619	0.308
Soil N	-0.30	-0.95	0.2072	0.019	0.32	-0.95	0.0106	0.827
Soil pH	0.63	-0.78	0.0515	0.435	-0.02	-1.00	0.0058	0.902
Soil water content	0.78	0.62	0.0416	0.553	-0.53	-0.85	0.0162	0.741

## Discussion

This study represents the first empirical work that demonstrates the wood-inhabiting fungal community assembly processes, including both stochastic and deterministic processes. This study has been carried out in tropical climate where the knowledge of wood-inhabiting fungi is highly limited [8]. Here we show that stochastic processes can also highly contribute to the wood-inhabiting fungal community assembly at both early and later stages of decomposing wood. Our work paves the way to a better understanding of wood-inhabiting fungal community assembly processes which are not solely shaped by deterministic processes or environmental filtering.

### Dynamics of the Stochastic and Deterministic Processes Changing with Time

After 2 years, the majority of deadwood samples reached decay class 3 which can be considered fast decomposition [32]. This can be explained by warm weather in this tropical forest and the relatively small size of wood materials [32, 53]. Opposite to what has been expected from the majority of studies that deterministic processes, especially environmental filtering through wood-physicochemical properties, control the community assembly of wood-inhabiting fungal communities [2, 9, 10], we found that both deterministic and stochastic processes can highly contribute to the community assembly processes of wood-inhabiting fungi in tropical forest [7]. Such contributions are highly depending on the decomposition stages of deadwood. The wood-inhabiting fungal community in deadwood at the initial stage is mainly governed by a deterministic process, especially homogenous selection whereas the early and later decomposition stages are governed by the stochastic processes, especially ecological drift. Opposite to the phenomenon observed in temperate forests [22, 23], we did not detect the priority effects in any tree species tested in this experiment carried out in tropical forests. Priority effect is known to mediate by intense biotic interactions (especially, biotic selection) by the initial species identities, and abundances affect subsequence species presence-absence and the community composition (effects of biotic interactions and dispersal on the presence-absence of multiple species). In our case, we found that the wood-inhabiting fungal communities show strong rearrangement patterns of fungal-fungal interactions (from modular structure to non-modular structure) and community composition after the deadwood is incorporated into soils. The strong degree of community turnover has been hypothesized to explain the absence of the priority effect of endophytes in deadwood decay [54]. We can clearly show that such rearrangements are mainly controlled by stochastic processes, especially ecological drift. The initial fungal community did not determine the subsequent communities, instead the dynamics of birth–death (i.e., random colonization-distinction) were important processes that regulate the community assembly of wood-inhabiting fungi in this tropical forest. It is noteworthy that the importance of ecological drift also increases over time from the early decomposition to later decomposition stages in both tree species tested in this study. Apart from homogenous selection and ecological drift, homogenized dispersal (stochastic process) was another important process that contributes to the assembly processes of wood-inhabiting fungi in this study. The contributions are consistent in both tree species and decomposition stages (mostly over 20%). This means in tropical ecosystems, some wood-inhabiting fungi have high dispersal rates; thus, they can colonize both favorable and non-favorable substrates and presence in all tree species and decomposition stages [7]. Consistent with this observation, we found a negligible impact of dispersal limitation (less than 2%) and variable selection (less than (4%) at local scale and only in *Q. pachyloma*). Such small contributions may be related to the specialist or red-list wood-inhabiting fungal taxa which have been reported to struggle with dispersal limitation [55]. High dispersal rates with low dispersal limitation have been reported before from temperate beech forests of Central Europe [56].

### Deterministic Processes Partly Shape Deadwood Mycobiome: the Importance of Wood Physicochemical Properties

Wood physicochemical properties have been hypothesized and reported to shape the wood-inhabiting fungal community composition [9, 10, 16]. We confirm here that the wood-physicochemical properties, especially hemicellulose content, C content, and alcohol-benzene extractives, consistently correspond with wood-inhabiting fungal community composition. In addition, N content, cellulose content, and pH also play an important role in shaping wood-inhabiting fungal community composition in *Q. pachyloma*. Macronutrients (such as C and N) as well as the major wood components such as cellulose and hemicellulose contents are important sources of precursors and/or energy used for fungal growth and reproduction. Anthropogenic disturbances such as thinning activities also contribute to shaping wood-inhabiting fungal community composition and richness in one tree species. High intensity of thinning has been reported in subtropical to reduce the fungal richness and alter their community composition [11]. It is interesting that all measured soil physicochemical properties, including macronutrients and pH, do not significantly shape the wood-inhabiting fungal community composition. Thus, our results suggest that the deterministic processes shaping deadwood mycobiome may arise from wood-physicochemical properties.

### Fungal-Fungal Interactions in Deadwood Strongly Differ in an Initial and Decomposing Deadwood

We observed the strong changes of network topology between the initial and 1-year decomposing deadwood from modular to non-modular communities. Later on, we also observed the modularity development between 1-year and 2-year decomposing wood. The first change may occur through the strong taxonomic and functional (from plant pathogens and/or mycoparasite to wood saprotrophs) differences between the initial and 1-year fungal communities. Different taxonomic and functional groups of wood-inhabiting fungi are found to differently interact with biotic and abiotic factors [2, 57]. The initial communities are mainly governed by deterministic processes which may be both attributed to biotic filtering (i.e., host tree species) and abiotic factors (environmental factors, wood traits, and wood physicochemical properties) [58]. In this current study, we also found that the initial fungal communities do not significantly shape the subsequent communities as the result of the priority effects or community assembly history. On the other hand, we found that stochastic processes (especially ecological drift) play an important role in controlling the transition of fungal communities from the initial deadwood to 1-year decomposing deadwood. Although there are significant changes of fungal taxonomic composition in deadwood samples collected 1 year as compared with 2 years, the dominant fungal functional group is wood saprotrophs. Thus, they may share some ecological attributes (especially their interaction with biotic and abiotic factors) and they may establish/develop co-occurring networks in the form of modular communities at this time [2, 58, 59]. Furthermore, due to the limitation of readily available resources at later stages of decomposition, fungi at this stage have been found to develop subcommunities that specialize for modified/degradation of complex substances such as lignin [60, 61]. We verify this assumption in our experiment as we detected a strong increase of Polyporales (with many members known as efficient lignin modifying/degrading enzyme producers) at the later stages of deadwood decomposition [62]. These Polyporales taxa interact with other members of Ascomycota and become important components of the subcommunities of wood-inhabiting fungi at the later stage of deadwood decomposition. In addition, we also demonstrate the functional (decay type) changes from soft-rot-dominated communities at the initial stages to soft- and white-rot communities after 1 year and finally to white-rot communities after 2 years. Soft-rot ascomycetes are known as pioneer species which partially degrade cellulose, hemicelluloses, and lignin under high moisture conditions were replaced by white-rot fungi which specialize in degrading all wood polymers, especially lignin [16, 63]. Brown-rot fungi’s ability to degrade hemicelluloses and cellulose but not or only partially lignin only co-occurred with low relative abundances in 2-year decomposing deadwood [63].

## Supplementary Information

Below is the link to the electronic supplementary material.

Supplementary file1 (XLSX 1072 KB)

Supplementary file2 (DOCX 53.6 KB)

## Data Availability

The fungal ITS raw read sequence datasets were deposited at the National Center for Biotechnology Information (NCBI) Sequence Read Archive (SRA) under bioproject number PRJNA887894.
