# Supplementary material for: Community Assembly Processes of Deadwood Mycobiome in a Tropical Forest Revealed by Long-Read Third-Generation Sequencing
Source: Microb Ecol. 2024 May 3;87(1):66. doi: 10.1007/s00248-024-02372-5 (PMC11068674; doi:10.1007/s00248-024-02372-5)
Supplement: Supplementary file 2 — Supplementary file2 (DOCX 53.6 KB) [file 248_2024_2372_MOESM2_ESM.docx]

**Supplementary Information**

**Title: Community assembly processes of deadwood mycobiome in a tropical forest revealed by long-read third-generation sequencing**

Witoon Purahong ^1*^, Li Ji^1, 2, *^ and Yu-Ting Wu^3,4†^,

**Affiliations:**

^1^UFZ-Helmholtz Centre for Environmental Research, Department of Soil Ecology, Theodor-Lieser-Str. 4, 06120 Halle (Saale), Germany

^2^School of Forestry, Central South University of Forestry and Technology, Changsha, 410004, China

^3^Department of Forestry, National Pingtung University of Science and Technology, Pingtung 91201, Taiwan

^4^Department of Biomedical Science and Environmental Biology, Kaohsiung Medical University, Kaohsiung 80708, Taiwan

**^*^**These authors contributed equally to this work.

^†^**Correspondence:**

Yu-Ting Wu: yutingwu@mail.npust.edu.tw

**Detailed protocols for wood-physicochemical properties**

**1. The equilibrium moisture content (EMC)**

EMC of both the heat-treated samples and the untreated control samples were determined according to the national standard for the determination of the moisture content for physical and mechanical tests (CNS452). Before the EMCs of the heat-treated samples were ascertained, the samples were stored in a conditioning room at 20°C and 65% relative humidity (RH) for approximately 4 weeks to reach equilibrium. Subsequently, the EMCs of the samples were calculated as follows:

| $\mathrm{EMC} (\%) = \frac{m_{u}－m_{o}}{m_{o}} \times100$ |  |
| --- | --- |

**2.** **The samples’ mass losses (MLs)**

MLs caused by the heat treatment were determined based on the oven-dried mass of the sample before treatment. The ML was determined as:

| $\mathrm{ML} (\%) = \frac{W_{o}－W_{1}}{W_{o}} \times100$ |  |
| --- | --- |

where *W_o_* is the oven-dried mass (g) of the sample before treatment, and *W_1_* is the oven-dried mass (g) of the same sample after heat treatment.

The national standard of the determination of density for physical and mechanical tests (CNS451) was applied to determine the densities of the heat-treated and control samples using the following equation:

| $\rho(kg/m^{3}) = \frac{m_{u}}{v_{u}}$ |  |
| --- | --- |

where *m_u_* and *v_u_* are the mass and volume, of the heat-treated or control sample, respectively, after conditioning.

**3. *pH***

Sample pH values were determined by shaking 1 g of dried wood in 10 mL of distilled water for 120 minutes and measuring the pH of the resulting aqueous extract.

**4. Cellulose and Hemicellulose**

After the wood sample is mill crushing and screening out the powder using mesh particle size ranging between 40 mesh (0.40 mm) and 60 mesh (0.246 mm) for the following analysis. According to CNS 4713 method, to wash the Soxhlet extractor, to weight after drying. After weighed the equivalent of the oven-dry weight of about 4 g of a sample placed in a cylindrical filter paper of Soxhlet extractor, followed by the addition of 150 ml Alcohol-benzene solution (Alcohol: benzene = 1: 2, volume ratio) and placed in a hot water bath for 6-8 h, siphon appears 6-8 times per hour, and then the solvent will be evaporated completely out of the bottle, the bottle is placed at 103 ± 2°C oven for 1 h, weight after cooling until the constant weight reaches, the following formula to calculate the percentage of absolute dry sample extraction.

Alcohol-benzene extracts (%) = W_a_/W_0_ x 100

W_a_： dried weight of extracts (g), W_o_： dry weight before extraction(g)

According to CNS 3085 for *holocellulose*, taking Alcohol-benzene extracts 2 g, placed in 250 mL Erlenmeyer flask, followed by adding 150 mL of distilled water, 0.2 mL glacial acetic acid and 1 g of sodium chlorite, a small 25 mL beaker covering the flask, the whole group place into 70-80°C water bath (Note water bath surface height is greater than the liquid surface in bottle), shake the bottle of liquid gradually turn golden yellow and divergent chlorine taste, from brown flour gradually turn yellow and white; to add 0.2 mL of glacial acetic acid and 1 g of sodium chlorite every 1 h, the reaction is for 5 h; and then the entire device move into an ice bath manipulation makes the temperature dropped to 10°C, 1G3 filter glass equipped with suction device, to wash the white residue on filters with 500 mL distilled water, and the filtrate cup is placed in original weighing bottle, placed at 60 ± 3°C suction (vacuum) to oven drying, weight after cooling, the following formula to calculate full-cellulose content.

$$\mathrm{Holocellulose}（\%）＝ \frac{W_{2}}{W_{0} / (1－W_{1})} \times100$$

W_0_：dried weight of extracts（g）, W_1_：Alcohol-benzene extracts (%)

W_2_：dried weight of residual holocellulose（g）。

According to CNS 10865 for *α-cellulose*, to weigh a 2 g sample in a 250 mL beaker, together with the beaker in a 20°C water bath, to add 15 mL 17.5% NaOH solution, the mixture is stirred with a glass rod for 1 min; add secondly 10 mL stirred for 45 sec; add thirdly 10 mL stirred for 15 sec after 3 min; finally to add 10 mL for 2.5 min, repeat four times, the total time is 10 min, cover with glass table stand for 30 min; add 100 mL distilled water and then thoroughly mixed for 30 min. To filter with a 1G3 filter cup, wash out the contents of the beaker. Stop sucking and rinse the beaker with 25ml of 8.3% NaOH solution, and then washed with 20^o^C 250 mL of distilled water in 5 times (50 mL × 5); Replace the 1G3 filter bowl suction device and continues rinsing with 20^o^C 400 mL distilled water, then stop pumping, adding 20^o^C of 2N acetate filter bowl filled with a glass rod was allowed to stand after stirring 5 min, the acetic acid removed by suction filtration, wash with distilled water at room temperature with a sufficient amount of acid in addition to remove the bowl is placed 103 ± 2^o^C oven drying to constant weight, the following formula to calculate α- cellulose content.

$$\alpha-cellulose（\%）＝ \frac{W_{2}}{W_{0} /W_{1}} \times100$$

W_0_：dried weight of holocellulose (g）, W_1_：Holocellulose (%)

W_2_：dried weight of residual α-cellulose（g）

    The method for measuring *semi-cellulose* will be followed by Zhao et al. (2010) with the following equation.

Hemicellulose content (%) = [holocellulose content (%)] – [α-cellulose content (%)]

**5. Acid insoluble lignin (Klason lignin)**

    According to CNS 2721 for Klason lignin (insoluble in acid), oven-dry weight of about 1 g ethanol - toluene extracts is placed in 100 mL beaker, and slowly adding 15 mL, 72% H_2_SO_4_ solution, and placed in 20°C water bath with frequent vortex this mixture, until fully gelled for about 2-4 h. Then wash thoroughly the gelled mixture with 560 mL of distilled water into a 1000 mL flask, equipped with a countercurrent condenser and heat to boil for 4 h. Finally, filter the mixture using a 1G3 glass filter cup and the residue is washed thoroughly with 500 mL of hot water, then put the bowl at 103°C ± 2°C oven drying to constant weight, and calculate its weight accounted for the one before ethanol extraction using the following formula.

$$lignin (insoluble in acid)（\%）＝ \frac{W_{2}}{W_{0} / (1－W_{1})} \times100$$

W_0_：dried weight of alcohol-benzene extracts（g）,

W_1_：Alcohol-benzene extracts (%)，

W_2_：Dried weight of residual lignin (insoluble in acid)（g）

According to CNS 3084 for ash, 40 mesh to 60 mesh wood powder is placed in a crucible. The crucible is placed into the preheated muffle fumace at 100°C. Lid is opened to avoid temperature rising rapidly which may cause flaming. It needs to be heat up slowly to 575°C and constantly open the lid up to have oxygen supplied until the temperature reaches 575°C and maintain the temperature for more than 3 h. Afterwards, transfer the crucible into the dessicator until cooling and weigh the sample.

$$Ash of wood (\%）＝ \frac{W_{1}}{W_{0}} \times100$$

W_1_：Ash of wood（g），W_0_：dried weight of sample（g）

**6. The analysis of carbon (C) and nitrogen (N)**

Elementar vario EL III CHN-OS Rapid F002 elemental analyzer was used for the analysis of carbon (C), and nitrogen element content. The ash content is determined using CNS 3084 method. The oxygen (O) (%) of the sample is calculated using the following formula.

$$O （\%）＝ 100\% －（C＋H＋N＋S＋\mathrm{ash}）\%$$

**Figure S1.** Average decay class (mean ± SE) of deadwood samples (*Quercus pachyloma* (Q) and *Machilus thunbergii* (M)) after 2 years.

**
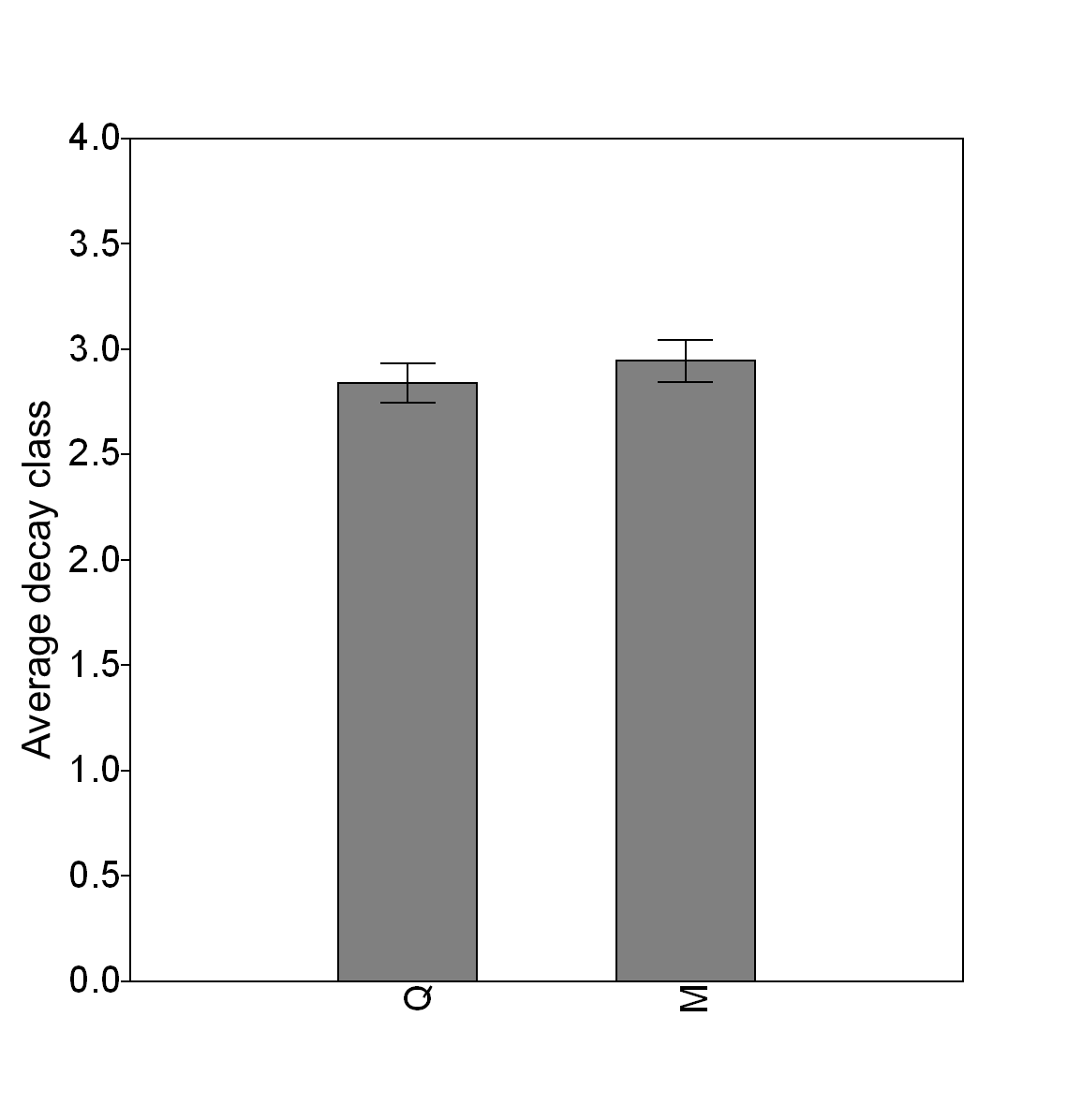
**

**Table S3.** Contributions of stochastic (HD, UP and DL) vs. deterministic processes (HS and VS) on wood-inhabiting fungal community assembly in deadwood of two tree species (*Quercus pachyloma* (Qu) and *Machilus thunbergii* (Ma)) at initial stage (Qu1 and Ma1), 1 year (Qu2 and Ma2) and 2 years (Qu3 and Ma3).

| BetaNTI/RCbray | Homogeneous selection | Variable selection | Homogenizing dispersal | Undominated processes | Dispersal limitation |
| --- | --- | --- | --- | --- | --- |
|  | HS | VS | HD | UP | DL |
| Qu1 | 48.53 | 2.21 | 25.53 | 22.65 | 1.08 |
| Qu2 | 21.21 | 0.00 | 22.68 | 56.11 | 0.00 |
| Qu3 | 13.68 | 3.68 | 20.11 | 60.77 | 1.75 |
| Ma1 | 39.47 | 0.00 | 26.12 | 34.40 | 0.00 |
| Ma2 | 15.79 | 0.00 | 22.65 | 61.56 | 0.00 |
| Ma3 | 6.32 | 0.00 | 9.86 | 83.82 | 0.00 |
